# Aging trends in skin cancer: A long-term observational study in Japan

**DOI:** 10.1016/j.jdin.2023.07.003

**Published:** 2023-07-22

**Authors:** Anna Nakamura, Kazuya Kataoka, Sumiko Takatsuka, Tatsuya Takenouchi

**Affiliations:** a Division of Dermatology, Niigata Cancer Center Hospital, Niigata, Japan; b Division of Dermatology, Niigata University Graduate School of Medicine and Dental Science, Niigata, Japan; c Department of Dermatology, Faculty of Medicine, Academic Assembly, University of Toyama, Toyama, Japan

**Keywords:** elderly cancer patients, Japanese, long-term observational study, nonmelanoma skin cancer, retrospective study, skin cancer

*To the Editor:* Incidence of skin cancer is increasing worldwide, particularly affecting the older population.^1^ This can be mainly attributed to increased cumulative ultraviolet exposure rather than other risk factors including exposure to ionizing radiation, immunosuppression, and long-term scar inflammation.^1^, ^2^, ^3^ The rapidly growing elderly population in Japan has led to an alarming increase in the number of skin cancer patients.^4^ However, studies comparing aging trends between patients with primary skin cancer and other organ cancers have not been reported. Herein, we conducted a long-term observational study focusing on the aging trends in skin cancer, at a tertiary cancer care hospital in Japan.

We retrospectively collected data of 5,669 patients who received treatment for primary skin cancer from 1989 to 2021 at the Niigata Cancer Center Hospital. The median age of skin cancer patients was observed to be 77 years (6-107), and included patients suffering from melanoma (*n* = 610), basal cell carcinoma (*n* = 1579), squamous cell carcinoma (*n* = 1161), Bowen's disease (*n* = 739), actinic keratosis (*n* = 994), extramammary Paget's disease (*n* = 210), skin appendage carcinoma (*n* = 215), and others (*n* = 161).

Fig 1 shows the age-wise distribution of the 5,669 skin cancer patients. The percentage of patients older than 70 years increased from 44% in 1989 to 74% in 2021. Of note, in the year 2021, 17% patients were older than 90 years of age. Fig 2 shows the change in median age of patients with melanoma (*n* = 561) and nonmelanoma skin cancer (NMSC, *n* = 4729) from 1991 to 2020, compared with primary other organ cancers such as the esophagus (*n* = 3280), stomach (*n* = 11,066), colon (*n* = 7525), liver (*n* = 1000), pancreas (*n* = 1820), lungs (*n* = 11,561), breast (*n* = 8785), uterus (*n* = 2778), ovary (*n* = 1099), prostate (*n* = 5363), urinary tract (*n* = 5349), and thyroid (*n* = 1842). In the past 5-year period, the median age of NMSC patients (80 years) was the highest of all, followed by that of liver cancer patients (73 years). The increasing rate of 16% for breast cancer was the highest, followed by liver cancer (14%) and NMSC (12%). The median age of melanoma patients did not show significant variations.

**Fig 1:**
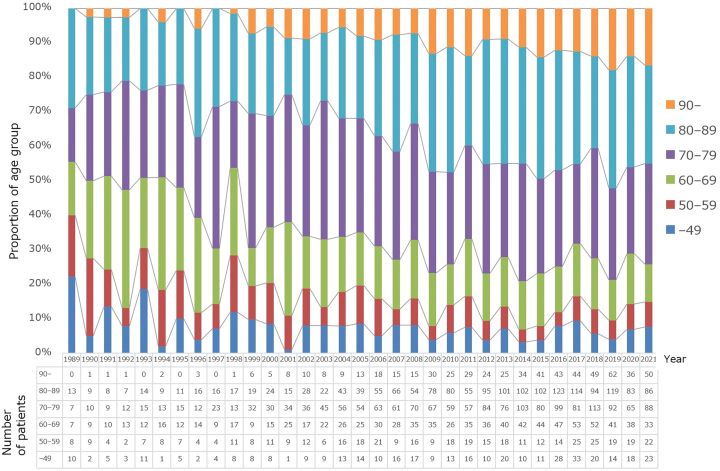
Variations in the age distribution of skin cancer patients. The graph shows trends in the proportions of age groups divided into 10-year intervals. The number of skin cancer patients increased from 45 in 1989 to 302 in 2021, with a particularly marked increase in patients aged 90 years old and above, accounting for 17% in 2021. The percentage of patients older than 70 years increased from 44% in 1989 to 74% in 2021.

**Fig 2:**
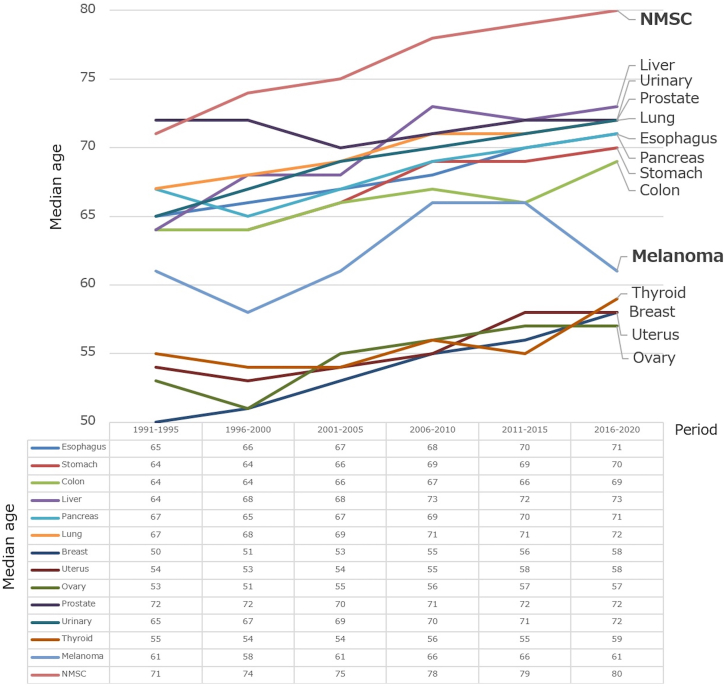
Changes in median ages of patients with major organ cancers. The graph shows changes in the median ages of major organ cancer patients divided into 5-year intervals from 1991 to 2020. Non-melanoma skin cancers (NMSC) showed more prominent aging trend than the other types of cancers.

The results of our study revealed the prominent aging trends in nonmelanoma skin cancer (NMSC) patients, compared to the other types of cancers. Our data is valuable for understanding long-term trends in NMSC, despite the study limitation from a single institution. The absence of aging trends in melanoma patients can probably be attributed to the high proportion of acral melanoma among Japanese patients.

Cancer treatment for elderly patients is complicated due to factors such as age-related decline in physical function, cognitive impairment, and the need for social care. Skin cancer in elderly patients is likely to be diagnosed at a more advanced stage, making the management more difficult.^5^ We need to consider educational strategies for early diagnosis and treatment targeting elderly patients.

## Conflicts of interest

None disclosed.
